# Studying Outcomes after Steroid-Sparing Immunosuppressive Agent vs. Steroid-Only Treatment for Immune-Related Adverse Events in Non-Small-Cell Lung Cancer (NSCLC) and Melanoma: A Retrospective Case-Control Study

**DOI:** 10.3390/cancers16101892

**Published:** 2024-05-16

**Authors:** Sharjeel Syed, Jacobi Hines, Rachel Baccile, Sherin Rouhani, Pankti Reid

**Affiliations:** 1 Department of Medicine, University of Chicago, Chicago, IL 60637, USA; sharjeel.syed@utsouthwestern.edu; 2 Division of Hematology-Oncology, Department of Medicine, University of Chicago, Chicago, IL 60637, USA; 3 Center for Health and The Social Sciences, University of Chicago, Chicago, IL 60637, USA; 4 Mass General Cancer Center, Massachusetts General Hospital, Boston, MA 02114, USA; 5 Division of Rheumatology, Department of Medicine, University of Chicago, Chicago, IL 60637, USA

**Keywords:** checkpoint inhibitors, immune-related adverse events, steroids, steroid-sparing immunosuppressive agents, melanoma, non-small-cell lung cancer

## Abstract

**Simple Summary:**

Corticosteroid use for the treatment of immune related adverse events (irAEs) after immune checkpoint inhibitor (ICI) use comes with concerns of side effects and the potential abrogation of antitumor immunity. Relatedly, the impact of steroid-sparing immunosuppressive agents (SSIAs) for treatment of irAEs on tumor outcomes is not well known, and current literature on this topic is limited by study design and issues around immortal time bias. This retrospective case-control study accounts for immortal time bias via strategic statistical methodology. Our results suggest that SSIAs used for irAE treatment may not negatively impact cancer outcomes in malignant melanoma and non-small cell lung cancer. For patients with melanoma, our results demonstrated better progression free survival for patients treated with infliximab compared to patients treated with corticosteroid monotherapy for irAEs. This study supports the safety for utilizing SSIAs for irAE treatment in patients receiving immune checkpoint inhibitors. Our study also encourages accounting for immortal time bias in the design of other observational studies in this space to accurately convey results and draw clinically meaningful conclusions.

**Abstract:**

Background: The effects of steroid-sparing immunosuppressive agents (SSIAs), used for the treatment of immune-related adverse events (irAEs), on immune checkpoint inhibitor (ICI) antitumor activity is not well known. We compared tumor outcomes of patients who received corticosteroid monotherapy (CS) versus a corticosteroid plus SSIA (CS-SSIA) for irAE treatment, using statistical methods to address immortal time bias. Methods: We conducted a retrospective case-control study on patients ≥ 18 years with melanoma or non-small-cell lung cancer (NSCLC) treated with ≥1 ICI at a quaternary care center between 1 January 2016 and 11 January 2021. Patients were divided into two cohorts: CS or CS-SSIA. We used propensity score nearest-neighbor matching to match on tumor type, stage, and prior lines of therapy. Primary outcomes were progression-free survival (PFS) and overall survival (OS). Secondary outcomes included the time from the start of the irAE treatment to the irAE resolution. Hazard ratios (HRs) for PFS and OS were calculated using the Cox proportional hazard regression method with both (1) the time to the steroid and SSIA as time-varying covariates and (2) a binary exposure classification not accounting for the time to the treatment. Results: A total of 167 patients were included after matching (132 in the CS cohort and 35 in the CS-SSIA cohort). Sixty-six percent of all the patients had melanoma. The most common irAEs requiring treatment were gastroenterocolitis and hepatitis. In an adjusted analysis not accounting for immortal time bias, there were no significant differences in PFS (HR 0.75, 95% CI [0.46–1.23]) or OS (HR 0.82, 95% CI [0.46–1.47]). In analyses using a time-varying treatment indicator, there was a trend toward improved PFS in patients treated with SSIAs (HR 0.54, CI 0.26–1.10). There was no difference in OS (HR 1.11, CI 0.55–2.23). Patients with melanoma who specifically received infliximab had improved PFS compared to patients with CS only, after adjusting for immortal time bias (HR 0.32, CI 0.24–0.43). Conclusions: The use of SSIAs with CS did not have worse outcomes than CS monotherapy. In melanoma, our findings showed improved PFS for the use of infliximab versus steroid monotherapy for irAEs. Large, prospective, randomized controlled trials are needed to confirm these findings and guide the optimal treatment of irAEs.

## 1. Introduction

Immune checkpoint inhibitors (ICIs) are used in a growing number of cancer types for both metastatic and early-stage cancers [1,2]. Currently approved ICIs block either cytotoxic T-lymphocyte-associated protein 4 (CTLA-4), programmed-cell-death protein/programmed death-ligand 1 (PD-1/PD-L1), or lymphocyte-activation gene 3 (LAG-3), and additional antibodies targeting other immune pathways are under investigation [2,3,4,5]. Although ICIs are generally well tolerated compared to traditional chemotherapy, they are frequently associated with immune-related adverse events (irAEs). The severity of irAEs is graded using the Common Terminology Criteria for Adverse Events (CTCAE). Grade 1 or 2 toxicity is seen in 50–60% of patients on single-agent PD-1 inhibitors, while more severe grade 3 or higher toxicity is only seen in approximately 20–30% of patients [6,7,8]. As the use of ICIs increases, increasing our understanding of the optimal management of associated irAEs is essential to maximize the potential benefit ICIs can offer to patients. 

In the past several years, many treatment guidelines have been formulated for the management of irAEs [9,10,11,12]. Recommendations are generally based on the impacted organ system(s), CTCAE grade, and the patient’s comorbidities. Immunosuppression remains the cornerstone of treatment, and high-dose corticosteroids are commonly used for first-line therapy. For many high-grade toxicities, guidelines recommend tapering corticosteroids over a 4–6-week period [6,9,10,11,12], leading to prolonged immunosuppression. However, despite these high-dose corticosteroids, the recurrence rate of irAEs is as high as 40%, and second-line immunosuppressive agents are frequently needed to manage high-grade toxicities [6,7,8,9,10,11,12,13]. Additionally, prolonged use of high-dose corticosteroids predisposes patients to serious infections due to non-specific immunosuppression, as well as metabolic derangements (e.g., new onset diabetes, adrenal suppression, weight gain, and hypertension), osteoporosis, gastritis, sleep disturbances, and neuropsychiatric adverse events [9,14,15,16,17,18]. 

Finally, when we are using steroids to ameliorate toxicities of effective immune-enhancing therapy against the patient’s cancer, mechanistically, there is concern that non-specific immunosuppression from glucocorticoids could mitigate the benefits of ICI therapy. This could detrimentally impact ICIs’ antitumor activity, subsequently impacting cancer outcomes [19,20]. A few retrospective studies on patients with melanoma or non-small-cell lung cancer have shown higher risks of death, shortened overall and progression-free survivals, and lower disease control rates for patients receiving corticosteroids [19,21,22,23,24]. However, follow-up studies conducting more robust subgroup analysis demonstrated that these outcomes were impacted by the indication for which corticosteroids were utilized; namely, when corticosteroids were used for palliative indications, patients had poorer outcomes, but when used for irAE treatments, this effect was not seen [19,24]. Many of the commonly used SSIAs have a narrower immunosuppressive profile compared to steroids and, therefore, may not have as much of an effect on tumor outcomes, but the effects on tumor outcomes have not been fully explored. 

Treatment for irAEs beyond steroids, with steroid-sparing immunosuppressive agents (SSIAs), is largely based on expert opinion and the treatment of similar autoimmune conditions in that organ system [9,10,11,12,25,26,27,28,29,30]. There remains limited evidence to guide recommendations regarding the timing and choice of SSIA(s) [9,10,11,12,26]. Recently, some groups have shown that introducing SSIAs upfront (as early as within 10 days of initiating steroid therapy) correlated with shortened steroid courses, decreased hospitalizations and infections, and quicker resolutions of irAEs [27,31,32,33]. Enhanced understanding of how the addition of SSIAs to corticosteroids (CSs) affects tumor outcomes could encourage clinicians to utilize SSIAs earlier in the treatment of severe toxicities. However, there is a paucity of information available about how SSIAs may impact cancer outcomes once immortal time bias is controlled for. Therefore, we conducted a retrospective case-control study on patients who received corticosteroid monotherapy (CSs) vs. corticosteroids + SSIAs (CS-SSIA) for the treatment of ICI-induced irAEs and used multiple statistical methods to account for immortal time bias. 

## 2. Methods

### 2.1. Study Design

A board-certified rheumatologist and oncologist identified 167 cases, which consisted of non-small-cell lung cancer (NSCLC) or melanoma patients ages ≥ 18 years who were treated with ≥1 ICI at a quaternary care center between 1 January 2016 and 11 January 2021. All the patients who were included had to have received treatment with systemic corticosteroids for irAE treatment at least 1 week prior to death. Patients who received only systemic corticosteroids for irAE treatment were assigned to the corticosteroid monotherapy (CS) cohort, while patients who also received SSIAs in addition to corticosteroids for irAE treatment were assigned to the corticosteroid + SSIA (CS-SSIA) cohort. The SSIAs of interest were limited to infliximab, tocilizumab, methotrexate, mycophenolate mofetil, rituximab, and tacrolimus.

### 2.2. Clinical Data Collection

Patient data were obtained from institutional electronic medical records in the University of Chicago Hospital’s system. Data on cancers, ICIs, irAEs, steroids, and SSIA-related information were extracted and confirmed in an iterative fashion by the investigators (S.S., J.H., S.R., and P.R.). Demographic data were extracted by adjunctive staff under the supervision of a physician. Diagnoses of irAEs were made by treating physicians. In cases of unclear irAE diagnosis and treatment, the investigators (a board-certified rheumatologist and oncologists) decided on inclusion vs. exclusion by group consensus. CTCAE v5.0 was utilized to grade the irAEs. Patients in the CS-SSIA cohort received corticosteroids first, and SSIAs were subsequently introduced usually because of corticosteroid treatment failure or intolerance. The time of the initiation and agent selection were determined by the treating medical team. Any infections that the patient developed during corticosteroid and/or SSIA treatment(s) were also documented from initiation to 30 days after the last dose of the last-used agent. The last date of the data abstraction was 30 June 2023. Finally, information on prior autoimmune history and steroid/SSIA use for autoimmune diseases was also documented.

### 2.3. Clinical Outcome Assessment

Primary cancer outcomes included progression-free survival (PFS), overall survival (OS), and cancer status. PFS was defined as the interval from the start date of the ICI therapy to the date of the progression; death due to any cause, if occurring before the progression; or the last follow-up, based on whichever was sooner. OS was defined as the interval from the start date of the ICI therapy to the date of the death; if patients were alive by the end of the study period, they were censored at the date of the last follow-up. The best overall response was assessed by oncologists (S.R. and J.H.) via the Response Evaluation Criteria in Solid Tumors (RECIST) version 1.1, and the timing of whether this occurred before or after irAE was noted. If the original radiographic scans were unavailable in the patient’s medical record, the treating oncologist’s documentation of the response was used when available. The date of the best response was assessed in a similar manner. If a patient received multiple lines of therapy with ICIs, the clinical outcome assessments were performed from the start of the ICI line, which resulted in irAEs requiring treatment with systemic steroids and/or SSIAs. Secondary outcomes included the time to the irAE resolution, defined as the sustained resolution of irAEs to grade 1 or lower, and irAE recurrence, defined as a recurrence in symptoms after 1 month of resolution.

### 2.4. Statistical Analysis

To construct comparable CS and CS-SSIA cohorts, propensity score full matching was implemented using the matchit function from the R MatchIt package [34]. A propensity score for SSIA treatment was estimated using logistic regression to regress an indicator variable for SSIA treatment based on the tumor type (melanoma or NSCLC), tumor stage (0–3 or 4), and number of prior treatment lines (0 or 1+). Full matching creates strata based on the propensity score of either one treated subject and at least one control subject or one control subject and at least one treated subject [35]. These strata are then weighted, allowing one to estimate the average treatment effect on the treated (ATT) with differently sized cohorts. Full matching enables the use of the full sample, avoiding bias due to incomplete matching and with other matching methods. It also allows for a larger effective sample size, thereby increasing the power [35].

We evaluated the effects of the immunosuppressive treatment on survival by comparing PFS and OS between the CS and CS-SSIA cohorts using Kaplan–Meier curves and Cox regression. A time-varying covariate for the treatment status from (1) the time from the ICI initiation to the time of the steroid initiation and (2) the time from the steroid initiation to the time of the SSIA initiation was included in the regressions to adjust for immortal time bias and non-proportional hazards. As a sensitivity analysis, we analyzed the following subgroups using the same methods as our main analyses: (1) infliximab vs. steroid only (both tumor types); (2) only melanoma, any SSIA vs. steroid only; (3) only NSCLC, any SSIA vs. steroid only; (4) only melanoma, infliximab vs. steroid only.

In our Cox regression models, we stratified by tumor type and stage to account for the differential underlying risks of progression and death. This estimates the ATT independent of tumor type and stage. 

All the analyses were conducted using two-sided tests; *p* values < 0.05 were considered as being significant. Categorical variables were described as propensity-score-weighted frequencies, and percentage and count variables were described as propensity-score-weighted medians with interquartile ranges. Balance was assessed using the standardized mean difference (SMD), with SMD > 0.10 indicating a meaningful difference; *p*-values were not used to assess balance, as advised in Ho et al. (2007) [34]. All the analyses were conducted in R, version 4.2.2, and used the survival package, version 3.4-0 [36,37]. Hazard ratios were reported along with robust 95% confidence intervals accounting for the stratification used with full matching. 

## 3. Results

### 3.1. Cohort Descriptive Comparisons

We used propensity score matching to exactly match patients based on tumor type, tumor stage, and number of lines of prior therapy. Before matching, our cohort was unbalanced based on age, tumor type, and single- vs. dual-agent ICI (Supplemental Table S1). After matching, our propensity-score-weighted cohort included 132 patients in the CS cohort and 35 in the CS-SSIA cohort. Four unmatched patients in the CS, who had stage III NSCLC and no prior lines of therapy, were excluded from the analysis. All the NSCLC patients with no prior lines of therapy in the CS-SSIA cohort were stage IV. 

### 3.2. Baseline Characteristics

Exact matching based on tumor type, stage, and the number of prior lines of therapy resulted in perfectly balanced cohorts based on those characteristics (Table 1). Despite not being included in the propensity score calculation, these cohorts are also well balanced based on age greater than or equal to 65, sex, race, autoimmune history, ECOG status, and single- vs. dual-agent ICI. Although the SMD of the age between the two cohorts was 0.28, the difference between the median age of 65 in the CS cohort and 62 in the CS-SSIA cohort is not clinically meaningful.

### 3.3. Tumor Outcomes: PFS and OS with and without Time-Varying Covariates

**Figure 1:**
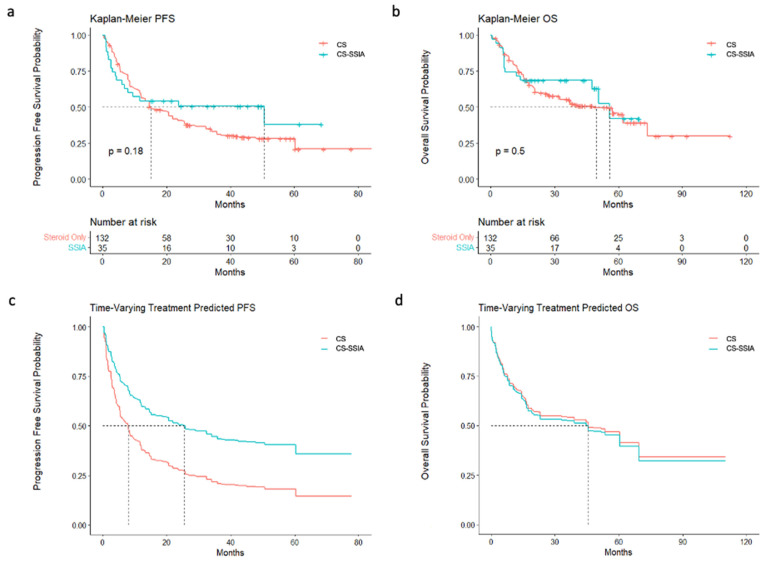
PFS and OS with and without time-varying covariate: (**a**) Kaplan–Meier curves of PFS for all the patients, stratified by CS vs. CS-SSIA. (**b**) Kaplan–Meier curves of OS for all the patients, stratified by CS vs. CS-SSIA. (**c**) Cox regression of PFS for all the patients, stratified by CS vs. CS-SSIA, accounting for time-varying covariate. (**d**) Cox regression of OS for all the patients, stratified by CS vs. CS-SSIA, accounting for time-varying covariate.

The models without a time-varying covariate for the treatment status did not indicate a statistically significant effect of the SSIA treatment on PFS or OS (Figure 1; Table 2). However, the Kaplan–Meier survival curves cross for both PFS and OS (Figure 1), indicating non-proportional hazards. Because of the non-proportional hazards resulting from immortal time bias, we also modeled survival with Cox regression, including a time-varying covariate for the treatment status. There was not a statistically significant association between any SSIA use and PFS (0.75, 95% CI [0.46–1.23]) or OS (0.82, 95% CI [0.46–1.47]) among our overall (melanoma + NSCLC) cohort (Figure 1; Table 2). This held true, even after accounting for immortal time biases for both PFS (0.54, 95% CI [0.26–1.10]) and OS (1.11, 95% CI [0.55–2.24]). Similarly, when the melanoma and NSCLC cohorts were analyzed separately, there were not any statistically significant associations between any SSIA use and PFS or OS.

We also conducted subgroup analyses evaluating survival differences between patients who received infliximab versus CS alone for irAEs (Table 2). There was a significantly lower PFS hazard ratio for infliximab use (0.46, 95% CI [0.22–0.97]) compared to patients who received CS only. This was the most pronounced amongst patients with melanoma treated with infliximab (PFS HR 0.32, 95% CI [0.24–0.43]) versus CS only. Our NSCLC infliximab cohort was too small (n = 3) to analyze separately.

### 3.4. Tumor Outcomes: The Best Overall Response

The CS-SSIA cohort had higher rates of patients with a complete response (Supplementary Table S2; 13.1% in CS vs. 37.1% in CS-SSIA, *p* = 0.01), and patients were more likely to have their best response after irAE compared to the CS cohort (22.2% in CS vs. 42.9% in CS-SSIA, *p* = 0.02). However, the CS cohort had higher rates of patients where the best overall response was unable to be assessed (Supplementary Table S2; 17.1% vs. 2.9%, *p* = 0.04)—this included cases where imaging was performed at an outside institution where the images or reports were not available for review, and there was no clear annotation of a response by the treating oncologist in the electronic medical record. The response was also unable to be assessed in cases in which the patient’s treatment changed prior to the first follow-up imaging. 

### 3.5. IrAE Baseline Information and IrAE Treatment Comparisons

The most common irAE requiring treatment among the CS-SSIA cohort was hepatitis (Table 3), which was significantly more common than in the CS cohort (20.9% in CS vs. 37.1% in CS-SSIA, SMD = 0.16). The most common irAE requiring treatment in the CS cohort was colitis (33.6%), which was equally common in the CS-SSIA cohort (31.4%). There were more patients in the CS-SSIA cohort with grade 3, 4, or 5 irAEs (59.9% vs. 81.8%, SMD = 0.22), and ICIs were less likely to be restarted after irAEs in the CS-SSIA group (26.8% in CS vs. 14.3% in CS-SSIA, SMD = 0.11). The most common used SSIAs were mycophenolate (42.9%) and infliximab (34.3%). The time to the irAE resolution was similar between the two groups (Table 4; 1.57 vs. 1.86 months, *p* = 0.27). There was a non-significant trend toward increased irAE recurrence rates in patients treated with CS alone (12.6% vs. 2.9%, *p* = 0.11). Rates of infections while patients were on immunosuppression drugs were similar between the two groups (11.3% vs. 20%, *p* = 0.22).

## 4. Discussion

Our results suggest that SSIAs do not have detrimental impacts on melanoma and NSCLC outcomes when utilized for the treatment of ICI-induced irAEs. In a subgroup analysis of patients with melanoma, PFS was better for patients treated with infliximab versus corticosteroid monotherapy for irAE management. 

These results are to be taken in the context of mixed evidence on this topic in the literature. A recent study by van Not et al. utilized a large Dutch registry of patients with melanoma to compare the impacts of CS vs. CS + SSIA on tumor outcomes. In this dataset, the receipt of SSIAs in addition to CS was associated with worse PFS (1.4, 95% CI [1.00–1.97]) and OS (1.54, 95% CI [1.03–2.30]) [38]. Alternatively, a review paper on TNF-alpha inhibition discusses preclinical and clinical data suggesting that TNF-alpha inhibitors are safe to use for irAE treatment; additionally, preclinical studies even show the potentially enhanced antitumor activities of these drugs blocking the TNF-alpha pathway [39,40]. Our results seem to be more aligned with these latter studies reflecting the safety of TNF-alpha inhibitors. Other steroid-sparing treatment options include the blockage of the interleukin-6 (IL-6) pathway, which has also been suggested to enhance antitumor activity [41]. 

Immortal time bias is one major potential confounding factor in retrospective irAE studies because clinically, SSIAs are generally added sequentially, if the irAE is refractory, to the initial treatment with corticosteroids. Patients must survive long enough to allow for SSIAs to be used; this lead time must be accounted for to accurately assess survival metrics. The importance for addressing immortal time bias in retrospective irAE research was illustrated in a comparison study published by Kfoury et al., where the development of an irAE was associated with improved survival when immortal time bias was accounted for with a time-varying Cox regression model but not with a landmark analysis [42]. There are minimal published data investigating how SSIAs impact cancer outcomes after accounting for immortal time bias [43]; therefore, we attempted to use robust statistical methods to address this question. 

Heterogeneity is also prevalent at multiple levels within studies reporting on outcomes after SSIA use for irAE treatment. For one, because of the absence of prospective trials investigating the optimal use of SSIAs in the treatment of irAEs, there remains notable variability in which agents are used, how early they are introduced, as well as the doses and scheduling that are utilized. The resultant variety in expert opinions and consequent differences in practice patterns makes comparing results across studies very difficult. Additionally, many studies looking at SSIA use for irAE treatment will incorporate multiple cancer types in the overall analysis, which if not adequately matched for, can make takeaways from PFS/OS results difficult to interpret, as there are significant inherent survival differences between tumor types. For example, the median PFS and OS for all comers with NSCLC and receiving chemoimmunotherapy are 9 months and 22 months, respectively; while in patients with malignant melanoma and receiving ipilimumab/nivolumab, the PFS and OS are 72.1 months and >78 months, respectively [44,45]. In this study, we controlled for this heterogeneity with propensity matching, with exact matches based on the tumor type, paired with stratified Cox regression. We were also able to conduct a subgroup analysis to observe differentiated results in the melanoma study population. 

Further confounding factors in this area of research arise from the manner in which SSIAs are introduced (often to treat irAEs that are steroid refractory) and the resultant difficulty in decoupling the impact and potential toxicities of SSIAs versus steroids. Often, not only have patients receiving SSIAs already been on steroids for long periods of time but also they continue to receive them simultaneously while SSIAs are introduced. The resultant overlapping timelines make delineating the impacts of one agent vs. another on PFS/OS, irAE resolution, cancer outcomes, and even certain side effects, difficult. Additionally, patients who do ultimately receive SSIAs are usually more likely to have received longer periods of systemic steroid exposure in the first place; indeed, our patients who received SSIAs, on average, had a steroid duration of 3.0 months compared to 2.2 months for the steroid-only group. This difference in steroid use between groups can lead to immortal time bias when analyzing SSIA vs. steroidal impacts, as the survival time between steroid and SSIA treatments is misattributed to the SSIA group. Similarly, immortal time bias can be seen in the period from the ICI treatment to the initiation of the first irAE treatment (which in our study, was always systemic steroids). The use of time-varying covariates, as in our study, can account for this bias and non-proportional hazards, unique to this area of research, and more accurately attribute PFS/OS times to exposure to steroids versus SSIAs. 

Ultimately, randomized controlled trials (RCTs) will be needed to overcome these potential confounding factors and definitively address the impacts of SSIAs on tumor outcomes. Several RCTs are currently underway testing whether the early introduction of SSIAs may improve irAE resolution or prevent irAE development. TNF-alpha inhibitors are frequently used for the second-line treatments of ICI-associated arthritis and ICI-associated colitis, and RCTs will test whether the early introduction of either infliximab in colitis (NCT05947669) or adalimumab in ICI-associated arthritis (NCT06037811) will shorten the duration of irAE symptoms and reduce steroid requirements. Although the primary outcomes of these trials are designed to measure the irAE improvement, they will also collect information about tumor outcomes, including PFS and OS. These trials may provide more evidence regarding the safety and efficacy for introducing TNF-alpha inhibitors earlier in the course of the irAE treatment. Another multicenter randomized trial (NCT05335928) is testing whether the early introduction of abatacept, a CTLA-4 agonist and T-cell inhibitor, improves major adverse cardiac event outcomes in patients with ICI-associated myocarditis. ICI-associated myocarditis is rare but has a very high mortality rate (up to 50%), highlighting the need for more effective treatment regimens. These and other RCTs will help to determine if SSIAs should be used upfront to gain control of irAEs more rapidly and potentially offer opportunities in the future to minimize the broader immunosuppressive effects of corticosteroids. There are also several other trials underway, which are testing the upfront use of SSIAs as a prophylactic measure to curb irAE development/severity and their potential to improve tumor outcomes in patients on ICIs. Preclinical studies using immune profiling identified increased levels of IL-6 in patients with ICI-associated enterocolitis, and blocking IL-6 led to enhanced antitumor responses in mouse models [41]. There are two trials that will test the addition of the IL-6 inhibitor tocilizumab to ipilimumab/nivolumab in patients with advanced melanoma to observe for differences in PFS/OS while monitoring the toxicity and development of adverse events (NCT03999749 and NCT04940299). Additionally, a phase I/II trial is underway testing the addition of tocilizumab to atezolizumab in advanced NSCLC as a means of improving the response rate and OS (NCT04691817). There is similar interest in the addition of B-cell-depleting medications, like rituximab, to limit irAE development and enhance ICI antitumor activity. The rationale for the former objective is based on the use of B-cell depletion to treat various immune-mediated inflammatory diseases and evidence that B-cell-associated antibodies are higher in patients developing various irAEs [46]. Additionally, a clinical trial testing the addition of the PD1 blockade to rituximab in relapsed follicular lymphoma was the first to show that the combination of these modalities could limit irAE development and improve response rates [47]. There is also a phase 2 randomized trial (NCT03719131) testing the addition of the CD20-depleting antibody rituximab to ipilimumab/nivolumab in patients with advanced melanoma. These trials will provide valuable insights into the potential impacts of SSIAs on antitumor activity while minimizing the confounding effects of steroids.

While we await the conduction of and results from these promising RCTs and other carefully designed prospective cohort studies, retrospective cohort studies with robust statistical approaches that account for immortal time bias can help to guide clinical practice. Our paper contributes to this field by providing a nuanced analysis on the impacts of specific classes of SSIAs on outcomes stratified by specific tumor types, with a decoupling between steroid and SSIA exposures via the careful consideration of time-varying covariates, propensity matching, and subgroup analysis. The limitations we faced include limits to power and generalizability, given our single-institution-based small sample size. As discussed above, like in other studies, we are limited by the provider’s experience in influencing the SSIA choice, which introduced considerable heterogeneity. We also had a significant number of patients excluded from the PFS analysis because of the unavailability of updated scans needed for a proper RECIST analysis, and the proportion of missing patient data was significantly higher in the steroid group, which limits the interpretation of our results for PFS. Nevertheless, our results suggest that the use of SSIAs does not worsen survival outcomes compared to CSs alone and that SSIAs should be used if necessary for irAE treatments.

## 5. Conclusions

Our study contributes to a growing body of data regarding the safety of SSIA use for ICI-induced irAE treatments in advanced melanoma and NSCLC. We also provide more clarity on improved methodological and statistical approaches to retrospective studies on these associations to better inform clinical practice while prospective studies and RCTs are underway. Still, there are many outstanding questions we hope that future studies can answer, including whether SSIAs could be used first line in lieu of systemic steroids and if particular classes of SSIAs may have improved tumor outcomes. As ICI use continues to expand to more patients and tumor types, optimal treatment strategies and standardized approaches to SSIA use will be more important than ever to improve outcomes for patients. 

## Figures and Tables

**Table 1:** Weighted baseline comparison of CS vs. CS-SSIA cohorts.

	CS	CS-SSIA	SMD
n	132	35	
Median Age at ICI Start	66 [57, 76]	62 [52, 70]	0.28
Age ≥ 65	61 (46.4)	18 (51.4)	0.05
Race			
Asian	1 (0.8)	1 (2.9)	0.02
Black	14 (10.8)	5 (14.3)	0.04
White	117 (88.4)	29 (82.9)	0.06
Sex			
Female	77 (58.4)	21 (60.0)	0.02
Male	55 (41.6)	14 (40.0)	
Tumor Stage			
II or III	26 (20.0)	7 (20.0)	0.00
IV	106 (80.0)	28 (80.0)	
Tumor Type			
Melanoma	87 (65.7)	23 (65.7)	0.00
NSCLC	45 (34.3)	12 (34.3)	
Has Prior History of Autoimmune Disease	5 (4.1)	1 (2.8)	
ECOG Performance Status			
0–1	122 (92.7)	34 (97.1)	0.04
2+	10 (7.3)	1 (2.9)	
Number of Prior Lines of Therapy			
0	91 (68.6)	24 (68.6)	0.00
1+	42 (31.4)	11 (31.4)	
Single-Agent vs. Dual-Agent ICI			
Single	82 (61.9)	19 (54.3)	0.08
Dual	50 (38.1)	16 (45.7)	

Notes: SSIA: steroid-sparing immunosuppressive agent; ICI: immune checkpoint inhibitor; IQR: interquartile range; ECOG: Eastern Cooperative Oncology Group. Counts for the CS cohort are rounded to the nearest whole integer after propensity score weighting; *p*-values were not calculated, as recommended by Ho et al. (2007). SMD > 0.10 indicates a statistically significant difference in characteristics between the groups.

**Table 2:** Progression-free and overall survival hazard ratios (HRs) of CS vs. CS-SSIA cohorts, with and without time-varying covariate.

	Progression-Free Survival	Overall Survival
	HR (95% CI)	HR (95% CI)
No Time-Varying Treatment	0.75 (0.46, 1.23)	0.82 (0.46, 1.47)
With Time-Varying Treatments: Melanoma and NSCLC, All the SSIAs	0.54 (0.26, 1.10)	1.11 (0.55, 2.24)
Melanoma and NSCLC: Infliximab Only vs. Steroids	0.46 (0.22, 0.97)	0.78 (0.28, 2.16)
Melanoma Only: All the SSIAs vs. Steroids	0.47 (0.21, 1.02)	0.89 (0.43, 1.82)
Melanoma: Infliximab Only vs. Steroids	0.32 (0.24, 0.43)	0.37 (0.14, 1.28)
NSCLC: All the SSIAs vs. Steroids	0.77 (0.32, 1.85)	1.84 (0.77, 4.43)

Notes: NSCLC: non-small-cell lung cancer. Models are stratified by tumor type and tumor stage; 95% confidence intervals are calculated using cluster-robust standard errors.

**Table 3:** Weighted comparison of irAE details in CS vs. CS-SSIA cohorts.

	CS	CS-SSIA	SMD
n	132	35	
**irAEs Requiring Treatment**, n (%)			
Colitis or Gastritis	44 (33.6)	11 (31.4)	0.02
Hepatitis	28 (20.9)	13 (37.1)	0.16
Pneumonitis	24 (18.2)	4 (11.4)	0.07
Myocarditis	7 (5.6)	2 (5.7)	0.00
Myositis	0 (0.2)	2 (5.7)	0.06
Dermatitis	24 (18.2)	3 (8.6)	0.10
Heme Toxicity	4 (3.0)	3 (8.6)	0.06
Nephritis	6 (4.8)	0 (0.0)	0.05
Neuro irAE	6 (4.2)	1 (2.9)	0.14
Arthritis	18 (13.3)	3 (8.6)	0.05
Others	7 (5.6)	2 (5.7)	0.00
**All irAEs Experienced**, n (%)			
Colitis or Gastritis	47 (35.7)	13 (37.1)	0.01
Hepatitis	33 (24.7)	15 (42.9)	0.18
Pneumonitis	25 (18.6)	4 (11.4)	0.07
Myocarditis	7 (5.6)	3 (8.6)	0.03
Myositis	1 (0.4)	2 (5.7)	0.05
Dermatitis	39 (29.7)	10 (28.6)	0.01
Heme Toxicity	4 (3.0)	3 (8.6)	0.06
Nephritis	6 (4.8)	0 (0.0)	0.05
Neuro irAE	6 (4.8)	1 (2.9)	0.02
Arthritis	20 (14.9)	4 (11.4)	0.04
Others	20 (15.2)	4 (11.4)	0.04
**Number of irAEs Per Patient**, n (%)			
1	76 (57.3)	18 (51.4)	0.06
2	39 (29.8)	9 (25.7)	0.04
3+	17 (12.9)	8 (22.9)	0.10
**Maximum CTCAE Grade of irAE Per Patient**, n (%)			
1–2	46 (40.1)	6 (18.2)	0.22
3–5	68 (59.9)	27 (81.8)	
**SSIA Used**, n (%)			
Mycophenolate Mofetil	-	15 (42.9)	-
Infliximab	-	12 (34.3)	-
Rituximab	-	3 (8.6)	-
Tocilizumab	-	2 (5.7)	-
Methotrexate	-	2 (5.7)	-
Tacrolimus	-	2 (5.7)	-
**Number of ICI Doses Before irAE**, Median [IQR]	3 [2, 8]	3 [2, 9]	0.10
**Patients Where ICI Was Restarted**, n (%)	35 (26.8)	5 (14.3)	0.11
**Months from ICI Start to irAE**, Median [IQR]	2.15 [1.15, 6.61]	2.33 [1.20, 6.70]	0.12
**Months from ICI start to Steroid Start**, Median [IQR]	2.99 [1.56, 7.84]	2.77 [1.23, 8.13]	0.06
**Months from ICI Start to SSIA**, Median [IQR]		3.54 [1.98, 10.30]	
**Total Duration of Steroid Treatment** (Months), Median [IQR]	2.17 [1.50, 4.00]	3.00 [2.00, 5.08]	0.18

Notes: SSIA: steroid-sparing immunosuppressive agent; ICI: immune checkpoint inhibitor; irAE: immune-related adverse event; IQR: interquartile range. Counts for the steroid-only cohort are rounded to the nearest whole integer after propensity score weighting; *p*-values were not calculated, as recommended by Ho et al. (2007). SMD > 0.10 indicates a meaningful difference.

**Table 4:** IrAE outcomes of CS vs. CS-SSIA cohorts.

	CS	CS-SSIA	*p*
n	132	35	
irAE Outcomes			
Did irAE recur?	17 (12.6)	1 (2.9)	0.11
Infection while on immunosuppression?			
No	117 (88.7)	28 (80.0)	0.22
Yes—on steroid or SSIA	15 (11.3)	7 (20.0)	
Yes—on steroid only	15 (11.3)	5 (14.3)	-
Yes—on SSIA +/− steroid	-	2 (5.7)	-
Time to irAE resolution, months (median [IQR])	1.57 [0.69, 3.06]	1.86 [1.01, 3.92]	0.27
Time to irAE resolution, data unavailable	14 (10.7)	8 (22.9)	0.09

Notes: SSIA: steroid-sparing immunosuppressive agent; irAE: immune-related adverse event; IQR: interquartile range; PFS: progression-free survival; OS: overall survival. Counts for the steroid-only cohort are rounded to the nearest whole integer after propensity score weighting.

## Data Availability

The authors confirm that the data supporting the findings of this study are available within the article and/or its Supplementary Materials. Raw data were generated at the University of Chicago Medical Center and can be available from the corresponding author (P.D.R.) upon reasonable request.

## Supplementary Materials

The following supporting information can be downloaded at https://www.mdpi.com/article/10.3390/cancers16101892/s1: Table S1: Baseline Comparison of Steroid Only vs. SSIA Cohort, Pre-matching; Table S2: Best Overall Response of CS vs. CS-SSIA cohorts.

## Abbreviations

ICIs	immune checkpoint inhibitors
irAEs	immune-related adverse events
SSIAs	steroid-sparing immunosuppressive agents
NSCLC	non-small-cell lung cancer
CS	corticosteroid monotherapy
CS-SSIA	corticosteroid + SSIA
