# Supplementary material for: Studying Outcomes after Steroid-Sparing Immunosuppressive Agent vs. Steroid-Only Treatment for Immune-Related Adverse Events in Non-Small-Cell Lung Cancer (NSCLC) and Melanoma: A Retrospective Case-Control Study
Source: Cancers (Basel). 2024 May 16;16(10):1892. doi: 10.3390/cancers16101892 (PMC11119129; doi:10.3390/cancers16101892)
Supplement: Supplementary file 1 [file cancers-16-01892-s001.zip › cancers-2979594-supplementary.pdf]

Supplemental Material

Supplemental Table S1. Baseline Comparison of Steroid Only vs SSIA Cohort, Pre-matching

	Steroid Only	SSIA/Steroid	SMD
n	136	35	
Median age at ICI start	68 [60, 76]	64 [52, 70.5]	0.43
Age >= 65	52 (38.2)	18 (51.4)	0.27
Race			
Asian	2 (1.5)	1 (2.9)	0.01
Black	23 (16.9)	5 (14.3)	0.03
White	111 (81.6)	29 (82.9)	0.01
Sex			
Female	77 (58.4)	21 (60.0)	118.00
Male	55 (41.6)	14 (40.0)	
Tumor Stage			
II or III	26 (20.0)	7 (20.0)	0.02
IV	106 (80.0)	28 (80.0)	
Tumor Type			
Melanoma	87 (65.7)	23 (65.7)	0.38
NSCLC	45 (34.3)	12 (34.3)	
Has prior history of autoimmune disease	5 (4.1)	1 (2.8)	0.02
ECOG performance status			
0-1	122 (92.7)	34 (97.1)	0.06
2+	10 (7.3)	1 (2.9)	
Number of prior lines of therapy			

0	91 (68.6)	24 (68.6)	0.05
1 +	42 (31.4)	11 (31.4)	
Single agent vs dual agent ICI			
Single	82 (61.9)	19 (54.3)	0.16
Dual	50 (38.1)	16 (45.7)	

Note: SSIA: Steroid-Sparing Immunosuppressive Agents; ICI: Immune Checkpoint Inhibitors; ECOG: Eastern Cooperative Oncology Group. P-values were not calculated as recommended by Ho et.al. (2007). SMD >0.10 indicates a meaningful difference.

Supplemental Table S2. Best Overall Response of CS vs CS-SSIA cohorts.

	CS	CS-SSIA	p
Best Overall Response			
Progression before irAE	10 (7.2)	5 (14.3)	0.23
Best overall response			
Complete Response or NED after surgical resection	17 (13.1)	13 (37.1)	0.01
Partial Response	32 (24.2)	7 (20.0)	0.63
Stable Disease	47 (35.8)	7 (20.0)	0.10
Progressive Disease	12 (9.3)	7 (20.0)	0.10
Unable to assess	23 (17.1)	1 (2.9)	0.03
Timing of best response*			
Prior to irAE; n (%)	80 (60.7)	29 (54.3)	0.51
After irAE; n (%)	29 (22.2)	15 (42.9)	0.02
Unable to determine	23 (17.1)	1 (2.9)	0.04

Note: SSIA: Steroid-Sparing Immunosuppressive Agents; irAE: Immune-Related Adverse Events; IQR: Interquartile Range; PFS: Progression Free Survival; OS: Overall Survival. Counts for the Steroid Only cohort are rounded to the nearest whole integer after propensity score weighting.
